# FLAIR-hyperintense lesions in anti-MOG-associated encephalitis with seizures overlaying anti-N-methyl-D-aspartate receptor encephalitis: A case report

**DOI:** 10.1097/MD.0000000000035948

**Published:** 2023-11-10

**Authors:** Qingxi Fu, Guangying Wang, Fengyuan Che, Dong Li, Shougang Wang

**Affiliations:** a Department of Neurology, Linyi People’s Hospital, Linyi, China; b Department of Neurology, Xuzhou Medical University, Xuzhou, China.

**Keywords:** anti-N-methyl-D-aspartate receptor encephalitis, cerebral cortical encephalitis, FLAIR-hyperintense lesions in anti-MOG-associated encephalitis with seizures, myelin oligodendrocyte glycoprotein, overlapping

## Abstract

**Rationale:**

FLAIR-hyperintense lesions in anti-myelin oligodendrocyte glycoprotein (MOG)-associated encephalitis with seizures (FLAMES) is a rare clinical phenotype of anti-MOG; immunoglobulin G-associated disease is often misdiagnosed as viral encephalitis in the early stages. Anti-N-methyl-D-aspartate receptor (NMDAR) encephalitis is an autoimmune encephalitis caused by antibodies targeting the GluN1 subunit of the NMDAR. The coexistence of anti-NMDAR encephalitis and FLAMES is very rare.

**Patient concerns:**

A 20-year-old female patient initially presented with seizures accompanied by daytime sleepiness.

**Diagnoses:**

Magnetic resonance imaging revealed FLAIR-hyperintense lesions in unilateral cerebral cortex. NMDAR antibodies was positive in the cerebrospinal fluid and MOG antibodies in the serum.

**Interventions:**

Steroid therapy was administrated.

**Outcomes:**

The symptoms completely relieved. At 6-month follow-up, the patient’s condition remained stable. Magnetic resonance imaging showed no abnormalities in the unilateral cerebral cortex.

**Conclusion:**

When a patient with anti-NMDAR encephalitis or FLAMES is encountered in clinical practice, the coexistence of these diseases with double-positive anti-NMDAR and MOG antibodies should be considered and adopt appropriate evaluation and treatment.

## 1. Introduction

Myelin oligodendrocyte glycoprotein (MOG) antibody-associated disease (MOGAD) is a MOG antibody-mediated idiopathic demyelinating disease of central nervous system (CNS), which is rare in both children and adults, with an incidence of 1.1 to 2.4 per million. It is seen more commonly in children than in adults, with an incidence of 3.1 per million.^[1]^ MOGAD presents with age-related clinical features: children patients mostly present with acute disseminated encephalomyelitis (ADEM)-like phenotypes (ADEM, ADEM with optic neuritis (ON), multiphasic ADEM, and encephalitis), while adults patients mostly present with optic nerve-spinal cord phenotypes (ON, myelitis) and brainstem encephalitis.^[1]^ MOG antibody-associated cerebral cortical encephalitis (CCE) is a rare type of MOGAD,^[2]^ and is less frequently reported in adults, which usually begins with seizures, headache and fever, and is often misdiagnosed as viral encephalitis in the early stage. FLAIR hyperintense lesions in anti-MOG-associated encephalitis with seizures (FLAMES) is a clinical phenotype of MOG antibody-associated CCE that has been reported since 2017.^[3]^

Anti-N-methyl-D-aspartate receptor (NMDAR) encephalitis is an autoimmune encephalitis (AE) mediated by antibodies targeting the GluN1 subunit of the NMDAR. Anti-NMDAR encephalitis is the most common AE subtype, accounting for approximately 54% to 80% of AE cases.^[4]^ It often presents with psychiatric/behavior abnormalities, seizures, and short-term memory loss. The coexistence of FLAMES and anti-NMDAR antibodies in 1 patient is very rare, which are collectively referred to as MOGAD and anti-NMDAR encephalitis overlapping syndrome (MNOS).^[5]^ Here we report a case of an adult patient with MNOS initially presenting with seizures, and review the relevant literature to improve our understanding of this disease.

## 2. Case presentation

A 20-year-old female patient was admitted to the hospital in August 2022 due to recurrent seizures for 1 month. One month ago, the patient suddenly developed tonic stiffening of the left extremities during a class, followed by tonic-clonic seizures of both extremities, lasting for about 2 minutes, then these symptoms were relieved. The patient underwent head computed tomography (CT) in local hospital and showed no abnormal findings, then she was treated with Piracetam. Five days later, the patient experienced daytime sleepiness and increased sleep need. Three days before admission to our hospital, the patient developed tonic-clonic seizures again during sleep in the early hours of the morning, lasting for about 2 minutes, which was then relieved, and accompanied by post-ictal headaches. Tonic-clonic seizures recurred during wakefulness in the afternoon (13:00) of the same day, which lasted for approximately 3 minutes and was relieved. She underwent head CT in local hospital again and showed no abnormal findings, then she was treated with Piracetam (0.8, 3 times a day). The patient had previously been in good health. She denied fever and infection, did not have history of head trauma or encephalitis, febrile seizures during childhood, alcohol or drug abuse, and had no family history of MNOS. Physical examination on admission revealed a body temperature of 36.2°C, a pulse rate of 83 beats/min, a respiratory rate of 19 breaths/min, and a blood pressure of 107/60 mm Hg. The patient had clear consciousness and normal speech. Results of neurologic examination of the cranial nerves, muscle strength, muscle tone, tendon reflexes of all 4 extremities were normal. Pathological reflexes were negative, sensory examinations were normal, with good movement coordination, and without signs of meningeal irritation.

**Figure 1.**
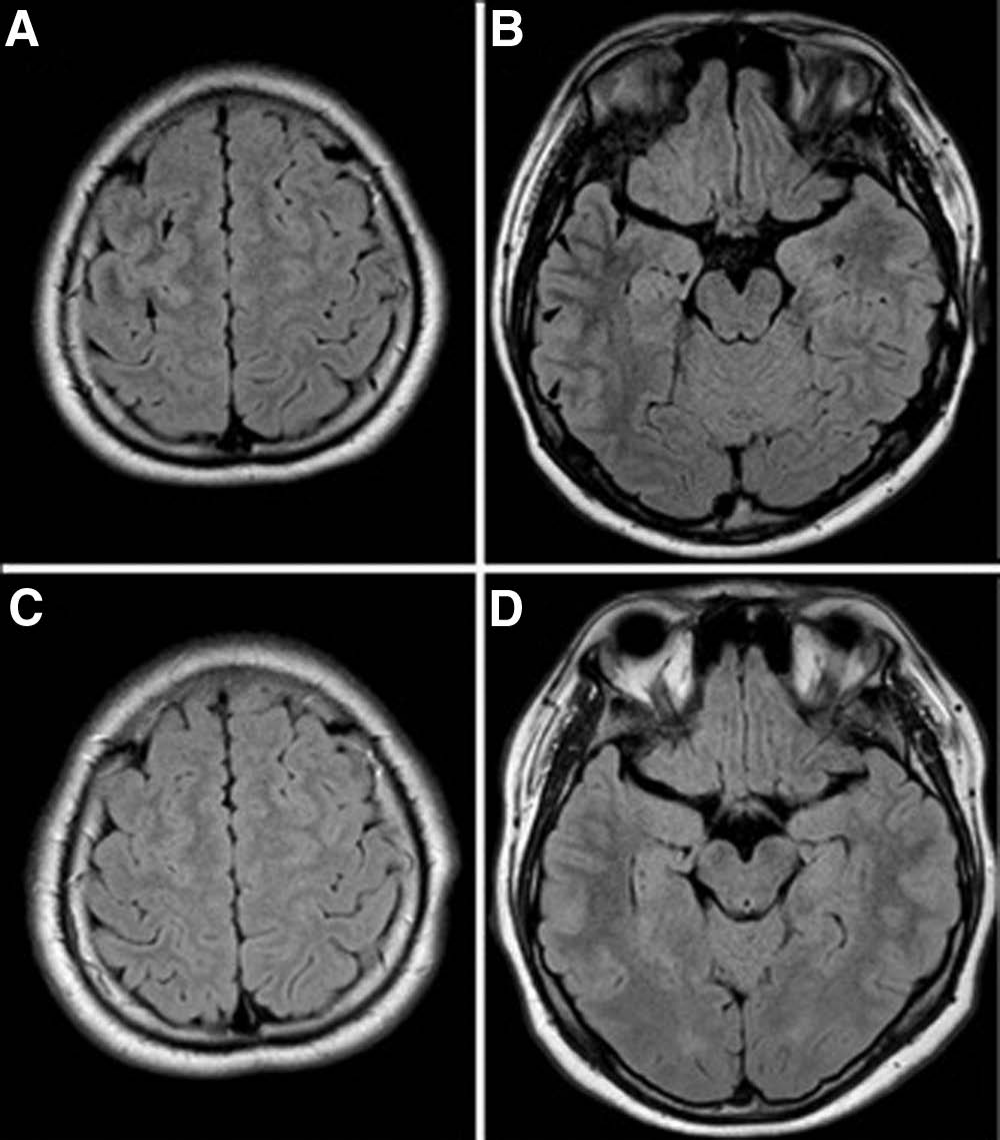
Brain MRI findings of the patient. (A and B) Brain MRI revealed slightly high signal intensity in the right frontal cortex, low signal on T2/FLAIR in right frontal subcortical white matter, and high signal on T2/FLAIR in the right temporal lobe. (C and D) Repeated FLAIR image showed that the low signal in the right frontal subcortical white matter had resolved, and the high signal in the temporal had disappeared after 6 months of treatment.

The blood, urine routine test results, C-reactive protein levels, blood glucose levels, liver and kidney function, blood coagulation function, blood lipids, thyroid function, infection screening results for AIDS, syphilis, hepatitis B, hepatitis C were both normal in the patient. She was negative for anti-neutrophil cytoplasmic antibodies, rheumatism antibody profile, and anticardiolipin antibodies. Chest CT revealed slight thickening of the lung texture bilaterally, and focal pleural thickening in the left pleura. Additionally, the abdominal ultrasound and gynecologic ultrasound were normal. Brain magnetic resonance imaging (MRI) revealed slightly high signal intensity in the right frontal cortex, low signal on T2/FLAIR in right frontal subcortical white matter, and high signal on T2/FLAIR in the right temporal cortex (Fig. 1A and B). Magnetic resonance angiography of the brain did not show any abnormalities. Electroencephalogram showed slow-wave activity in the right frontal and temporal regions.

Cerebrospinal fluid (CSF) examination revealed a pressure of 260 mmH_2_O, a protein concentration of 245 mg/L, a glucose level of 3.13 mmol/L, a chloride level of 123 mmol/L, and cell count of 18 × 10^6^/L. Cytology showed a large number of inflammatory cells, without the presence of tumor cells. Next-generation sequencing of RNA and DNA extracted from CSF of the patient did not identify any significant pathogenic microorganisms. Cell-based assay showed that the patients were tested positive for anti-NMDAR-IgG antibodies in her CSF (titer: 1:10), and MOG antibodies in the serum (titer:1:32).

The patient was treated with methylprednisolone (1 g/d) for 5 days, followed by maintenance therapy with oral administration of prednisone acetate tablets (60 mg/d, 1 tablet was reduced every 2 weeks). Meanwhile, antiepileptic drug treatment with oral administration of levetiracetam (500 mg, twice daily) was given. Daytime sleepiness resolved after 1 month of treatment, and seizures did not occur over the entire treatment period. At a follow-up after 6 months of treatment, the patient was asymptomatic. MRI with epilepsy-specific sequences showed that the low signal in the right frontal subcortical white matter had resolved, and the high signal in the temporal had disappeared (Fig. 1C and D). Repeated examinations of serum NMDAR and MOG antibodies showed negative results. The patient was satisfied with the treatment results and her recovery.

## 3. Discussion

MOG is a glycoprotein exclusively expressed on the surface of the oligodendrocytes and myelin sheaths in the CNS. MOG antibody positivity can be seen in inflammatory demyelinating diseases of the CNS, which is currently known as MOGAD.^[6]^ FLAMES is a rare clinical phenotype of MOGAD. In this study, the patient presented initially with seizures. MRI showed focal high signal intensity on T2/FLAIR in the right frontal cortex and temporal cortex, low signal on T2FLAIR in the subcortical white matter. These MRI findings were consistent with the imaging features of MOG antibody-associated CCE observed in a previous study.^[7]^ And the patient was tested positive for serum MOG antibodies. Although the clinical manifestations of MOGAD, including ON and myelitis were absent in the patient, the diagnosis of FLAMES was considered.

Anti-NMDAR encephalitis is a treatable AE with the presence of antibodies that react specifically with the NR1 subunit of NMDAR. It is the predominant type of AE, and usually presents with psychiatric/behavior abnormalities, seizures, speech disorders, movement disorders, memory deficits, sleep disorders.^[8]^ In present study, the patient met the diagnostic criteria for AE proposed by Graus et al.^[9]^ She presented with seizures and sleep disorders. MRI revealed focal gyral swelling of the right temporal lobe with inflammatory changes. The video-electroencephalogram revealed localized abnormal spike-and-slow wave discharges. CSF was positive for anti-NMDAR antibodies.

Clinically, double positivity for anti-NMDAR and MOG antibodies is very rare, with an incidence of <10% in patients with anti-NMDAR encephalitis or MOGAD.^[10]^ The incidence of NMDAR positivity in patients with MOG antibody-associated CCE is slightly higher (13%).^[7]^ Double antibody positivity can occur sequentially or simultaneously. The mechanism for the simultaneous coexistence of these antibodies is unclear. It has speculated that abnormal immunity may attack the autoantigens of MOG and NMDAR in the same location, and lead to the generation of antibodies that induce the development of diseases.^[5]^ In addition, viral infection may be the predisposing factor for the coexistence of anti-NMDAR and MOG antibodies.^[11]^ Upon infection with neurotropic viruses, the blood–brain barrier is disrupted, the antigens of MOG and NMDAR leak into the peripheral blood, followed by the activation of helper T cells and the recruitment and activation of MOG- and NMDAR-specific B cells, eventually leading to the production of anti-MOG and anti-NMDAR antibodies. However, in present study, CSF examination did not show evidence of viral infection.

Clinically, patients who are double positive for both anti-NMDAR and MOG antibodies exhibit symptoms of both anti-NMDAR encephalitis and MOGA, with convulsions, mental and behavioral abnormalities being as the common symptoms in the acute stage. Patients can present either with typical symptoms of anti-NMDAR encephalitis (such as psychiatric/behavior abnormalities, seizures, short-term memory loss, speech disorder, motor deficits, decreased level of consciousness, and autonomic dysfunction), or symptoms of MOGDA (such as headache, fever, ON, ataxia, and dystonia), or typical symptoms of both diseases. However, in contrast to patients with typical anti-NMDAR encephalitis, patients with MNOS show milder symptoms of encephalitis.^[12]^ In terms of treatment, steroid therapy is the first-line treatment option for both anti-NMDAR encephalitis and MOGAD in the acute stage. Clinically, it has been proven that most patients improve after immunotherapy, but a consensus has not been reached regarding the duration of steroid therapy. In this study, the patient showed relatively mild clinical symptoms. The reasons may be that the patient had relatively weak antibody titers, she was diagnosed with MNOS earlier, and treated with steroid therapy in a timely manner.

In conclusion, when a patient with anti-NMDAR encephalitis or FLAMES is encountered in clinical practice, the possibility of coexistence of these diseases, with double-positive anti-NMDAR and MOG antibodies in the CSF and serum, should be considered. The appropriate antibodies should be selected for testing to determine the patient’s condition as early as possible and adopt appropriate evaluation and treatment. Additionally, available data on MNOS have shown that the recurrence rate of MNOS was close to that of MOGA.^[13]^ MOG antibodies may play an important role in the recurrence of MNOS. Therefore, for patients who have been diagnosed with MNOS, regular monitoring of serum MOG antibody levels during the follow-up period is of great importance.

## Author contributions

**Conceptualization:** Shougang Wang.

**Investigation:** Qingxi Fu, Guangying Wang, Fengyuan Che, Dong Li.
